# Toward the Pathogenicity of the *SLC26A4* p.C565Y Variant Using a Genetically Driven Mouse Model

**DOI:** 10.3390/ijms22062789

**Published:** 2021-03-10

**Authors:** Chin-Ju Hu, Ying-Chang Lu, Ting-Hua Yang, Yen-Hui Chan, Cheng-Yu Tsai, I-Shing Yu, Shu-Wha Lin, Tien-Chen Liu, Yen-Fu Cheng, Chen-Chi Wu, Chuan-Jen Hsu

**Affiliations:** 1 Department of Otolaryngology, National Taiwan University Hospital, Taipei 100, Taiwan; ginniehu@g.harvard.edu (C.-J.H.); lu16889@gmail.com (Y.-C.L.); thyang37@ntu.edu.tw (T.-H.Y.); f93548030@gmail.com (Y.-H.C.); leon9139@gmail.com (C.-Y.T.); liuent@ntu.edu.tw (T.-C.L.); cjhsu@ntu.edu.tw (C.-J.H.); 2 Program in Speech and Hearing Bioscience and Technology, Harvard Medical School, Boston, MA 02115, USA; 3 Department of Medical Research, Taipei Veteran General Hospital, Taipei 112, Taiwan; 4 Department of Otolaryngology, Taichung Tzu Chi Hospital, Buddhist Tzu Chi Medical Foundation, Taichung 427, Taiwan; 5 Transgenic Mouse Models Core (TMMC), Division of Genomic Medicine, Research Center for Medical Excellence, National Taiwan University, Taipei 100, Taiwan; isyu@ntu.edu.tw (I-S.Y.); mtshuwha@ntu.edu.tw (S.-W.L.); 6 Department of Otolaryngology-Head and Neck Surgery, Taipei Veteran General Hospital, Taipei 112, Taiwan; 7 Faculty of Medicine, National Yang Ming Chiao Tung University, Taipei 112, Taiwan; 8 Institute of Brain Science, National Yang Ming Chiao Tung University, Taipei 112, Taiwan; 9 Department of Medical Genetics, National Taiwan University Hospital, Taipei 100, Taiwan; 10 Department of Otolaryngology, College of Medicine, National Taiwan University, Taipei 100, Taiwan

**Keywords:** *SLC26A4*, p.C565Y variant, Pendred syndrome, mice, human

## Abstract

Recessive variants of the *SLC26A4* gene are globally a common cause of hearing impairment. In the past, cell lines and transgenic mice were widely used to investigate the pathogenicity associated with *SLC26A4* variants. However, discrepancies in pathogenicity between humans and cell lines or transgenic mice were documented for some *SLC26A4* variants. For instance, the p.C565Y variant, which was reported to be pathogenic in humans, did not exhibit functional pathogenic consequences in cell lines. To address the pathogenicity of p.C565Y, we used a genotype-based approach in which we generated knock-in mice that were heterozygous (*Slc26a4^+/C565Y^*), homozygous (*Slc26a4^C565Y/C565Y^*), and compound heterozygous (*Slc26a4^919-2A>G/C565Y^*) for this variant. Subsequent phenotypic characterization revealed that mice with these genotypes demonstrated normal auditory and vestibular functions, and normal inner-ear morphology and pendrin expression. These findings indicate that the p.C565Y variant is nonpathogenic for mice, and that a single p.C565Y allele is sufficient to maintain normal inner-ear physiology in mice. Our results highlight the differences in pathogenicity associated with certain *SLC26A4* variants between transgenic mice and humans, which should be considered when interpreting the results of animal studies for *SLC26A4*-related deafness.

## 1. Introduction

Recessive variants of the *SLC26A4* (Gene ID: 5172) gene are a common global cause of hereditary hearing impairment (HHI) [1]. In certain populations, pathogenic *SLC26A4* variants can be identified in approximately 15–20% patients with HHI [2]. *SLC26A4* encodes for pendrin, a chloride bicarbonate transporter that is mainly expressed in the thyroid, inner ears, kidneys, lungs, liver, and heart [3,4]. Recessive *SLC26A4* variants cause the occurrence of Pendred syndrome (PS; MIM #274600) and nonsyndromic hearing loss, DFNB4 (MIM #600791). DFNB4 is characterized by isolated sensorineural hearing impairment (SNHI), which is usually associated with a common inner-ear malformation known as enlarged vestibular aqueduct (EVA; MIM 603545), whereas patients with PS have goiter in addition to EVA [5]. Clinically, patients with *SLC26A4* variants either with the manifestation of DFNB4 or PS usually suffer from progressive or fluctuating SNHI [6].

To date, more than 400 *SLC26A4* variants were reported (http://deafnessvariationdatabase.org/ (accessed on 30 June 2020). Corresponding with the extensive variation in the severity of clinical phenotypes, different *SLC26A4* variants are associated with different pathogenic consequences in cell-line experiments [7,8]. Some variants, such as p.H723R, p.L236P, and p.T721M, are associated with defective protein expression or trafficking, which leads to the accumulation of pendrin in either the cytoplasm or the perinuclear region. Some variants, such as p.K369Eand p.S166N, are associated with the normal protein expression of pendrin in the cell membrane, but impaired protein function for chloride bicarbonate (Cl^–^/HCO_3_^–^) exchange [7,8]. Interestingly, an enigmatic *SLC26A4* variant, p.C565Y, was previously reported as pathogenic in humans when present in trans with other *SLC26A4* mutations, such as p.Q514R [9,10], p.L236P [11], and p.H723R [12]. However, p.C565Y produced minimal or no functional pathogenic consequences in the HEK 293 and COS-7 cell lines or *Xenopus* oocytes [8,9]. Therefore, it is crucial to clarify the discrepancy in the pathogenicity between humans and cell lines in order to dissect the pathogenic mechanisms related to *SLC26A4* variants.

In addition to cell-line models, transgenic mice were also demonstrated to be a powerful tool for investigating the pathogenic mechanisms of *SLC26A4* variants [13,14,15,16,17,18,19]. However, previously established mouse models with different *SLC26A4* variants were reported to have diverse auditory phenotypes, ranging from normal to profound hearing loss, none of which perfectly recapitulated the clinical phenotypes in patients. In this study, we generated a knock-in mouse model harboring the p.C565Y variant of *Slc26a4,* and investigated the associated audiovestibular phenotype and inner-ear pathology. We also generated compound heterozygous mice (*Slc26a4^919-2A>G/C565Y^*) by intercrossing p.C565Y mice with *Slc26a4^919-2A>G/919-2A>G^* mice. The latter is a transgenic strain previously established in our laboratory with confirmed abolished *Slc26a4* function. Heterozygous mice were used to clarify the pathogenicity of p.C565Y in monoallelic, biallelic, or compound heterozygous forms.

## 2. Results

### 2.1. Gross Inner-Ear Morphology

**Figure 1:**
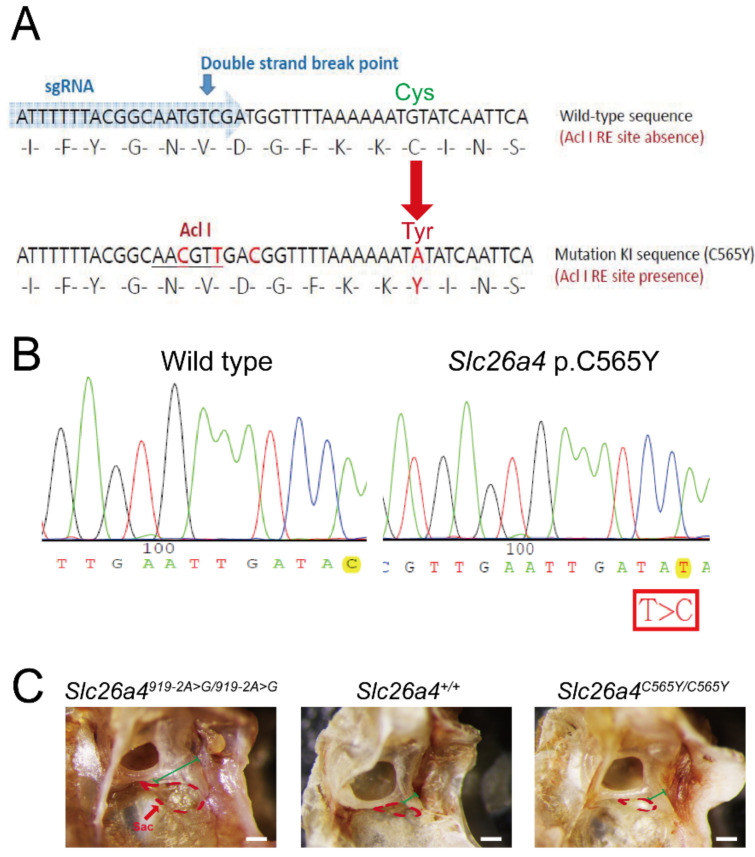
Generation of mice with *Slc26a4* p.C565Y variant using clustered regularly interspaced short palindromic repeat (CRISPR)/Cas9. (**A**) Design diagram. Single guide RNA (SgRNA) for CRISPR/Cas9 and silent mutations for enzyme cutting sites (for checking) were designed to incorporate mutations into the genome of C57BL/6 mice. To generate the *Slc26a4* p.C565Y variant, codon TGT was mutated to TAT. (**B**) Sanger sequencing was performed to confirm nucleotide changes in transgenic mice. Sequence was read in reverse. (**C**) Gross morphology. Enlargement in endolymphatic sac (region delineated by red dotted line) and vestibular aqueduct (marked by green line) was observed in the *Slc26a4^919-2A>G/919-2A>G^* mice, but not in *Slc26a4^+/+^* and *Slc26a4^C565Y/C565Y^* mice (bar = 100 μm).

Previous studies revealed that pathogenic *Slc26a4* variants could cause enlarged endolymphatic sac, dilated vestibular aqueducts, and inflated scala media [18,20]. Enlargement of the endolymphatic sac and vestibular aqueducts was observed in *Slc26a4^919-2A>G/919-2A>G^* mice, but not in *Slc26a4^+/+^* and *Slc26a4^C565Y/C565Y^* mice (Figure 1C).

### 2.2. Auditory-Phenotype Evaluation and Cochlear Histology

**Figure 2:**
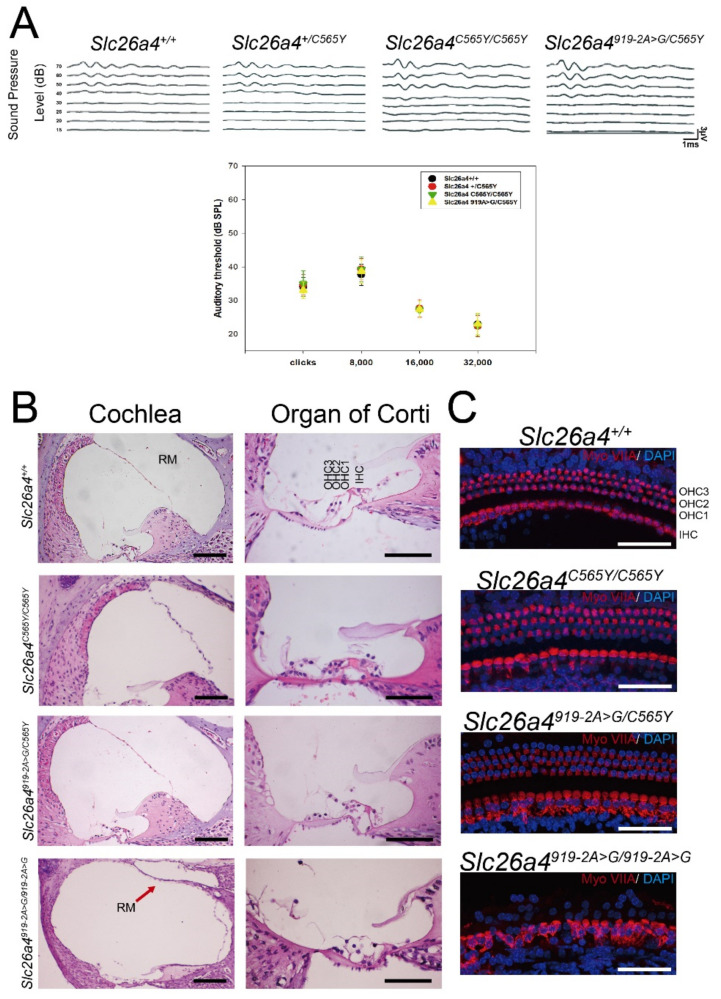
Auditory phenotypes and cochlear histology. (**A**) Heterozygous *Slc26a4^+/C565Y^*, homozygous *Slc26a4^C565Y/C565Y^*, and compound heterozygous *Slc26a4^919-2A>G/C565Y^* mice showed normal hearing thresholds across different frequencies, similar to that of wild-type *Slc26a4^+/+^* mice. (**B**) Cochlear histology harvested at 3 months. Abnormal histological phenotypes observed in *Slc26a4^919-2A>G/919-2A>G^* mice, including dilatation of scala media (Cochlea panel; RM, Reissner’s membrane) and degeneration of cochlear hair cells (Organ of Corti panel), which were not observed in *Slc26a4^+/+^*, *Slc26a4^C565Y/C565Y^*, and *Slc26a4^919-2A>G/C565Y^* mice (bar = 150 μm). (**C**) Histology of cochlear hair cells harvested from mice at three months. Myosin-VIIA expression was normal in *Slc26a4^+/+^, Slc26a4^C565Y/C565Y^*, and *Slc26a4^919-2A>G/C565Y^* mice when compared to diminished expression in *Slc26a4^919-2A>G/919-2A>G^* mice (bar = 50 μm).

Wild-type (i.e., *Slc26a4^+/+^*), heterozygous (i.e., *Slc26a4^+/C565Y^*), and homozygous (i.e., *Slc26a4^C565Y/C565Y^*) mice (*n* = 10 each) were subjected to audiological evaluations at P28 (Figure 2A). Both *Slc26a4^+/C565Y^* and *Slc26a4^C565Y/C565Y^* mice exhibited no significant differences in hearing compared to *Slc26a4^+/+^* mice at up to 9 months (Supplementary Figure S1), indicating that the p.C565Y allele is not causal for SNHI in mice.

To confirm the pathogenicity of the p.C565Y allele in mice, we further generated compound heterozygous mice (i.e., *Slc26a4^919-2A>G/C565Y^*) upon intercrossing *Slc26a4^+/C565Y^* mice with *Slc26a4^919-2A>G/919-2A>G^* mice [15]. Similar to heterozygous mice with c.919-2A > G (i.e., *Slc26a4^+/919-2A>G^*), *Slc26a4^919-2A>G/C565Y^* mice (*n* = 10) also exhibited no significant difference in hearing compared to *Slc26a4^+/+^* mice up to 9 months. This finding indicates that the p.C565Y allele is nonpathogenic and that the single p.C565Y allele is sufficient to maintain auditory function in mice.

Cochlear histology was also investigated in homozygous (i.e., *Slc26a4^C565Y/C565Y^*) and compound heterozygous (i.e., *Slc26a4^919-2A>G/C565Y^*) mice. For this, the cochleae of wild-type mice and profoundly deaf *Slc26a4^919-2A>G/919-2A>G^* mice were obtained for comparison. Abnormal histological phenotypes observed in *Slc26a4^919-2A>G/919-2A>G^* mice, including the dilatation of scala media (Figure 2B) and degeneration of cochlear hair cells (Figure 2B), were not observed in *Slc26a4^+/+^, Slc26a4^C565Y/C565Y^*, and *Slc26a4^919-2A>G/C565Y^* mice. Fluorescence confocal-microscopy analysis revealed that, unlike in *Slc26a4^919-2A>G/919-2A>G^* mice, cochlear hair cells were not degenerated or disorganized in *Slc26a4^+/+^, Slc26a4^C565Y/C565Y^*, and *Slc26a4^919-2A>G/C565Y^* mice (Figure 2C).

### 2.3. Vestibular-Function Evaluation and Histology of Vestibular Organs

**Figure 3:**
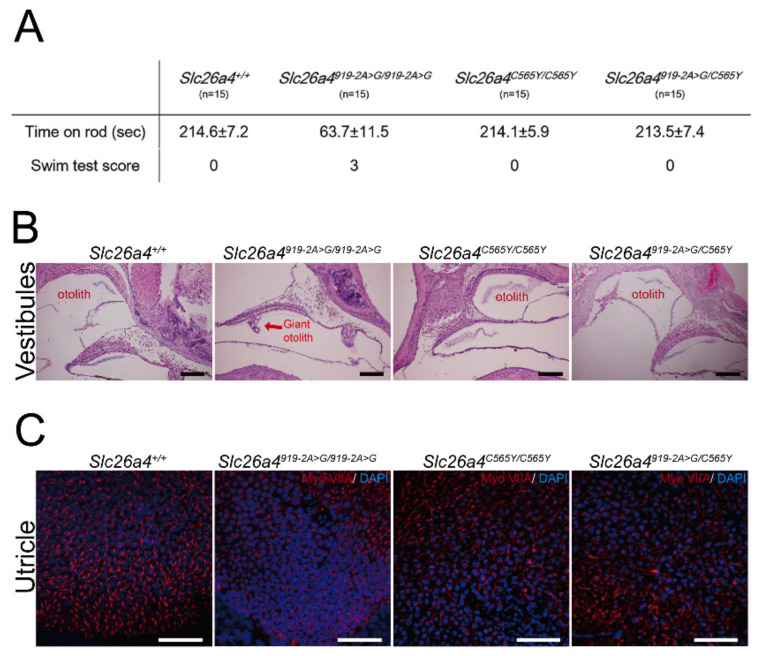
Vestibular phenotypes and histology of vestibular organs. (**A**) In contrast to *Slc26a4^919-2A>G/919-2A>G^* mice, homozygous *Slc26a4^C565Y/C565Y^* mice and compound heterozygous *Slc26a4^919-2A>G/C565Y^* mice performed well in swimming and rotarod tests, similar to wild-type *Slc26a4^+/+^* mice. (**B**) Histology of vestibular organs. Giant otoconia observed in *Slc26a4^919-2A>G/919-2A>G^* mice, but was normal in *Slc26a4^+/+^, Slc26a4^C565Y/C565Y^*, and *Slc26a4^919-2A>G/C565Y^* mice (bar = 150 μm). (**C**) Fluorescence confocal microscopy. In contrast to *Slc26a4^919-2A>G/919-2A>G^* mice, vestibular hair cells in *Slc26a4^+/+^, Slc26a4^C565Y/C565Y^*, and *Slc26a4^919-2A>G/C565Y^* mice did not degenerate (bar = 50 μm).

A total of 60 mice, namely, 15 *Slc26a4^+/+^*, 15 *Slc26a4^919-2A>G/919-2A>G^*, 15 *Slc26a4^C565Y/C565Y^*, and 15 *Slc26a4^919-2A>G/C565Y^* mice, were subjected to vestibular-function evaluation. Similar to the normal audiological phenotypes, homozygous (i.e., *Slc26a4^C565Y/C565Y^*) and compound heterozygous (*Slc26a4^919-2A>G/C565Y^*) mice did not exhibit vestibular deficiencies such as head-tilting and circling behavior. Moreover, both of these strains performed normally in the swimming and rotarod tests (Figure 3A). These findings indicate that the single p.C565Y allele is sufficient to maintain normal vestibular function in mice.

Giant otoconia in vestibular organs (Figure 3B) and the degeneration of vestibular hair cells (Figure 3C) were observed in *Slc26a4^919-2A>G/919-2A>G^* mice, whereas *Slc26a4^+/+^*, *Slc26a4^C565Y/C565Y^*, and *Slc26a4^919-2A>G/C565Y^* mice exhibited normal histology for vestibular organs (Figure 3B,C).

### 2.4. Morphology of Stria Vascularis

**Figure 4:**
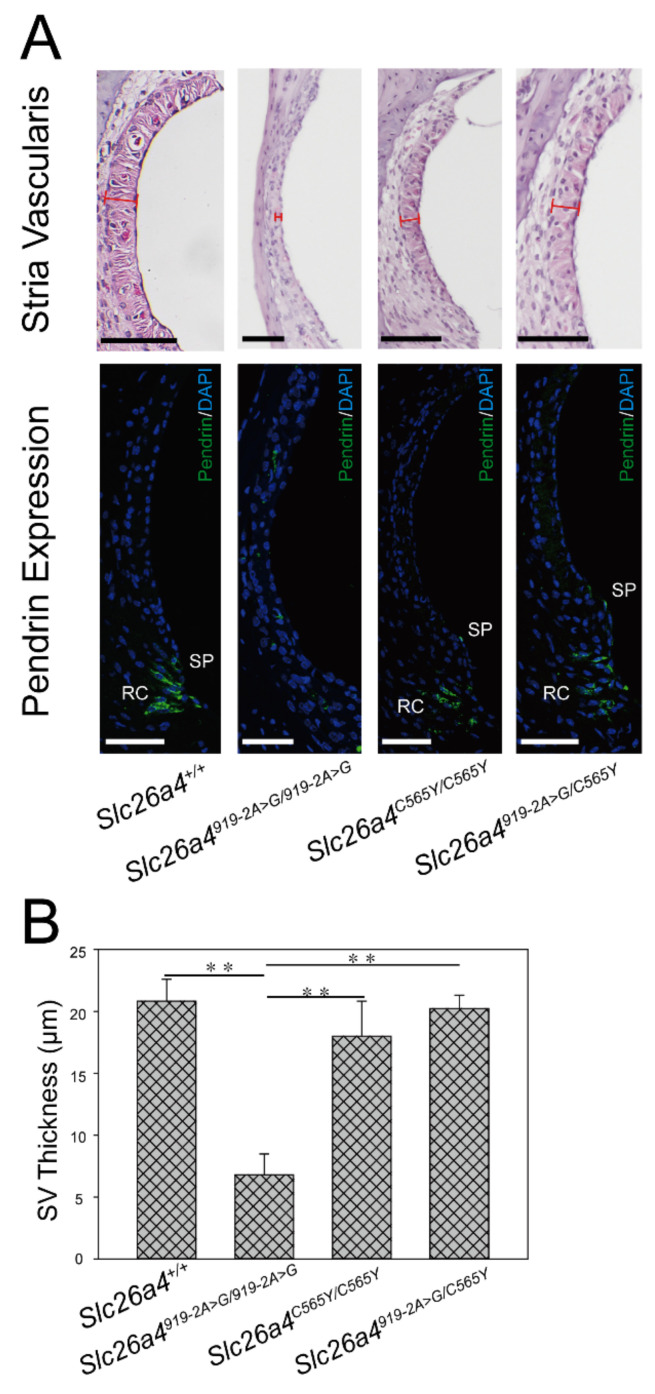
Morphology of stria vascularis (SV) and pendrin expression. (**A**) Histology and expression of pendrin in stria vascularis. Significant atrophy of SV and poor protein expression of pendrin were observed in *Slc26a4^919-2A>G/919-2A>G^* mice. By contrast, pendrin was normally distributed in spiral prominence (SP) and root cells (RC) in *Slc26a4^+/+^, Slc26a4C^565Y/C565Y^*, and *Slc26a4^919-2A>G/C565Y^* mice, similar to in that of wild-type mice. Tissue samples harvested from mice at 3 months (bar = 50 μm). (**B**) Quantification data of SV thickness. SV thickness in each group of mice was calculated (*n* = 3). In *Slc26a4^+/+^, Slc26a4^C565Y/C565Y^*, and *Slc26a4^919-2A>G/C565Y^* mice, SV was thicker than that of *Slc26a4^919-2A>G/919-2A>G^* mice, in which SV was atrophic (** *p* < 0.01).

Atrophic stria vascularis (SV) with decreased thickness was documented in mice with various pathogenic *Slc26a4* variants [15,21,22,23]. Significant atrophy of SV was observed in *Slc26a4^919-2A>G/919-2A>G^* mice, but not in *Slc26a4^+/+^, Slc26a4^C565Y/C565Y^*, or *Slc26a4^919-2A>G/C565Y^* mice (Figure 4A). There was significant difference in SV thickness among the four groups (Figure 4B, *n* = 3 for each group).

The expression of pendrin in the cochlea of *Slc26a4^C565Y/C565Y^* and *Slc26a4^919-2A>G/C565Y^* mice was examined by immunolocalization assay (Figure 4A). In both strains, pendrin was normally distributed in root cells and the apical membranes of spiral prominence surface epithelial cells, as in wild-type mice (*Slc26a4^+/+^*) [24], indicating that the expression of pendrin was unaffected in mice with the p.C565Y variant.

## 3. Discussion

In this study, we generated a transgenic mouse model harboring the p.C565Y variant of the *Slc26a4* gene to examine the pathogenicity of the p.C565Y variant in mice. Phenotypic characterization revealed that both homozygous (i.e., *Slc26a4^C565Y/C565Y^*) and compound heterozygous (i.e., *Slc26a4^919-2A>G/C565Y^*) mice exhibited hearing and balance comparable to those of wild-type mice, which was further confirmed by normal inner-ear morphology in histological studies. These findings indicate that the p.C565Y variant of *Slc26a4* gene is nonpathogenic for mice.

The *SLC26A4* p.C565Y variant was documented as a pathogenic variant in several previous reports, where p.C565Y was exclusively present in trans with another *SLC26A4* mutation, including p.Q514R [9,10], p.L236P [11], and p.H723R [12]. With the aid of the Varsome platform [25], which combines a human genetic-frequency database (e.g., gnomAD), a disease variant database, (e.g., Clinvar, Uniprot), and many predictive algorithms to assess variant pathogenicity, p.C565Y was categorized as a likely pathogenic variant in accordance with criteria of the American College of Medical Genetics and Genomics (ACMG) guidelines for PM1, PM2, PM5, PP2, PP3, and PP5 (see definitions, explanations, and classifications in Tables S1–S3, respectively). In addition to being classified as a pathogenic variant by many predictive algorithms (SIFT, DANN, EIGEN, FATHMM-MKL, MutationTaster, and others) and databases that include ClinVar, DVD, and Uniprot, p.C565Y also demonstrated low allelic frequency (AF) across populations (Popmax Filtering AF < 0.0001 in GenomAD) and location at a critical domain of pendrin where pathogenic *SLC26A4* variants were recurrently detected. Therefore, the pathogenicity of the *SLC26A4* p.C565Y variant in humans can be confirmed by several lines of evidence classified in the ACMG guidelines (Supplementary Tables S1–S3), indicating difference between humans and mice.

The mutation landscape of the *SLC26A4* gene significantly differs among different ethnic groups [12,26,27,28]. Previous clinical studies presented inconsistent results in regard to the correlation between *SLC26A4* genotypes and phenotypes [29,30,31]. In several studies, the authors did not identify any phenotypic differences among patients with different *SLC26A4* variants [32,33]. However, some variants might lead to the progression of hearing loss [34,35], and some *SLC26A4* genotypes may be associated with a normal thyroid phenotype and less severe hearing loss [36]. The p.C565Y variant was clinically detected in three patients. All three segregated p.C565Y in the trans configuration with another *SLC26A4* variant. One patient harbored the p.Q514R/p.C565Y genotype. This patient was diagnosed as having Pendred syndrome and bilateral EVA, goiter, and abnormal perchlorate discharge value [9,10]. The patient with the p.L236P/p.C565Y genotype also suffered from goiter, abnormal perchlorate discharge value, and early childhood SNHI [11]. The final patient with the p.C565Y/p.H723R genotype was diagnosed as nonsyndromic EVA without thyroid manifestation [12]. Otherwise limited clinical information makes it difficult to delineate a clear phenotype that is specifically associated with the p.C565Y variant.

Consistent with these recent clinical reports, studies based on cell-line experiments demonstrated that different *SLC26A4* variants have different pathogenic mechanisms. Some variants, such as p.H723R, p.L236P, and p.T721M, are associated with defective protein expression or trafficking, whereas some other variants, such as p.K369E and p.S166N, are associated with normal protein expression but impaired protein function [7,8]. Notably, even among each subgroup, a gradient of pathogenicity appears to exist. For instance, the trafficking of pendrin in the p.H723R variant could be rescued by salicylate and temperature manipulation, whereas pendrin in the p.L236P variant could not be rescued. The p.C565Y variant seems to be nonpathogenic in cell lines, as pendrin with p.C565Y is expressed correctly in the plasma membranes in both HEK 293 [7,8] and COS-7 cell lines [9], and is also shown to exhibit normal protein function in *Xenopus* oocytes [9].

Similarly, different *SLC26A4* variants are associated with different phenotypic severities in animal models. To date, several mouse models were generated, including knock-out *Slc26a4^−/−^* mice [13], *Slc26a4^loop/loop^* mice with the p.S408F variant [14], *Slc26a4^919-2A>G9/919-2A>G^* mice with the c.919-2 A>G variant [15], *Slc26a4^H723R/H723R^* mice with the p.H723R variant [16], *Slc26a4^L236P/L236P^* mice with the p.L236P variant [17], conditional knock-outs Tg[E]; Tg[R]; *Slc26a4*^Δ/Δ^ mice [18], and hH723R Tg mice with the p.H723R mutation in the context of the human pendrin protein [19] (Table 1). *Slc26a4^−/−^*, *Slc26a4^loop/loop^*, hH723R, and *Slc26a4^919-2A>G9/919-2A>G^* mice exhibited congenital profound SNHI, which represents the most severe symptom in the phenotypic spectrum, whereas *Slc26a4^H723R/H723R^* mice exhibited normal hearing, representing the least severe symptom in the phenotypic spectrum. *Slc26a4^L236P/L236P^* and Tg[E]; Tg[R]; *Slc26a4*^Δ/Δ^ mice revealed an intermediate phenotype, such that some residual hearing could be recorded in both strains. 

Interestingly, there seems to be some correlation between pathogenicity in cell lines and animals. Variants with stronger pathogenicity in cell lines, such as p.L236P [7,17], correlate with the presence of phenotypes in transgenic mice, whereas variants with weaker or no pathogenicity in cell lines, such as p.C565Y [8], correlate with the absence of phenotypes in transgenic mice. This correlation might be helpful for researchers selecting *SLC26A4* variants as potential subjects when generating transgenic mouse models.

Despite the presence of these correlations in the pathogenicity of *SLC26A4* variants, cell-line and transgenic mouse experiments still possess some limitations that preclude them from being satisfactory experimental models for *SLC26A4*-related SNHI. First, some variants that are supported by strong evidence to be clinically pathogenic, such as p.K369E and p.C565Y, did not reflect any abnormality in protein expression or function in cell lines [8,9,37], or audiovestibular phenotypes in transgenic mice [16]. The trafficking or glycosylation process might also significantly differ between mice and humans [38], which could explain the absence of phenotypes in mice with the p.C565Y and p.H723R variants of the *Slc26a4* gene. Alternatively, as p.C565Y was documented as pathogenic in humans only when present in trans with other *SLC26A4* variants, such as p.Q514R, p.L236P, and p.H723R [9,10,11,12], it cannot be excluded that p.C565Y homozygosity alone is nonpathogenic. An extensive study combining these other variants may be necessary for a comprehensive understanding of the pathogenicity of the p.C565Y variant, considering that human clinical data are limited. Second, the amino acid sequence of pendrin differs across species. For instance, the amino acid sequence of the pendrin C terminus (i.e., amino acids 508–780) shares only 86% identity between mice and humans (https://www.expasy.org/ (accessed on 18 August 2020), which might explain the inconsistent pathogenicity of p.C565Y between mice and humans. To address this, humanized transgenic mice generated with mutant human cDNA sequences could provide a solution, as this approach can better recapitulate clinical phenotypes in humans [19,39]. Third, even for transgenic mice with positive phenotypes, there are still valid discrepancies in the audiovestibular features that are reported between mice and humans. For instance, the congenital profound SNHI reported in *Slc26a4^−/−^*, *Slc26a4^loop/loop^*, hH723R, and *Slc26a4^919-2A>G9/919-2A>G^* mice is too severe when compared to their phenotypes in human counterparts. Although *Slc26a4^L236P/L236P^* and Tg[E]; Tg[R]; *Slc26a4*^Δ/Δ^ mice showed some residual hearing, neither could perfectly recapitulate the fluctuating or progressive disease nature in humans. Further studies are required to refine cell-line or transgenic-mouse models to investigate and better understand *SLC26A4*-related SNHI.

In conclusion, we generated a knock-in mouse model that segregated the deafness-associated *SLC26A4* p.C565Y variant in humans in a genotype-driven approach. Subsequent phenotypic characterization revealed that mice with the *Slc26a4* p.C565Y variant exhibit normal audiovestibular phenotypes and inner-ear morphology, indicating that the pathogenicity associated with specific *SLC26A4* variants might differ between humans and mice. Therefore, with regard to research on *SLC26A4*-related SNHI, caution should be taken when translating results of animal studies to humans.

## 4. Materials and Methods

### 4.1. Generation of Slc26a4^C565Y/C565Y^ Knock-In Mice

Transgenic mice were generated using the Transgenic Mouse Models Core (TMMC, Taiwan) and the clustered regularly interspaced short palindromic repeat (CRISPR) technology-associated RNA-guided endonuclease Cas9 to mutate the *Slc26a4* gene and further generate the *Slc26a4^+/C565Y^* mouse strain. Specific guide RNAs (sgRNAs) were developed to target exon 15 of the *Slc26a4* gene in the C57BL/6 mouse strain. The sgRNA and CRISPR/Cas9 RNA were delivered simultaneously into the zygote of a C57BL/6 mouse to generate the founders. Two male founder mice were obtained, each possessing the p.C565Y (c.1694G>A) variant in the *Slc26a4* gene. After germline transmission of the targeted mutation allele, we were able to generate the congenic *Slc26a4^+/C565Y^* mouse line by repeated backcrossing with the C57BL/6 inbred strain for 6–10 generations. Thereafter, the homozygous mice for the *Slc26a4*p.C565Y variant (*Slc26a4^C565Y/C565Y^*) were obtained by intercrossing heterozygous mice (*Slc26a4^+/C565Y^* × *Slc26a4^+/C565Y^*; Figure 1A,B).

Corresponding to the human genotypes, compound heterozygous mice (i.e., *Slc26a4^919-2A>G/C565Y^*) with both the p.C565Y and c.919-2A>G variants were also generated by intercrossing heterozygous *Slc26a4^+/C565Y^* mice with *Slc26a4^919-2A>G/919-2A>G^* mice [16]. Mice were housed at the National Taiwan University College of Medicine Laboratory Animal Center. The mice were bred and weaned at approximately 3 weeks of age in a vivarium with an alternating 12 h light/dark cycle at controlled temperature (20–26 °C) and humidity (40–70%). Bedding was refreshed once a week. Water and food were always available. All animal experiments complied with animal-welfare guidelines and were approved by the Institutional Animal Care and Use Committee (IACUC) of the National Taiwan University College of Medicine (approval no. 20160337, 1 November 2016). For euthanasia, we intraperitoneally injected barbiturate (100 mg/kg) into each mouse and performed cervical dislocation. All experiments were performed without regard to the sex of mice, and mice were randomly selected for every independent measurement. In total, 75 mice were used in this study, including *Slc26a4^+/+^*, *Slc26a4^C565Y/C565Y^*, *Slc26a4^919-2A>G/C565Y^*, and *Slc26a4^919-2A>G/919-2A>G^*.

### 4.2. Auditory Evaluations

For audiological evaluations, 28 day postnatal mice (P28) were intraperitoneally anesthetized with pentobarbital (35 mg/kg; Sigma-Aldrich, St. Louis, MO, USA) and maintained in an acoustically and electrically insulated, and grounded test room [16]. The thresholds of the auditory brainstem response (ABR) in mice were measured with an evoked potential detection system (Smart EP 3.90; Intelligent Hearing Systems, Miami, FL, USA). Click sounds and 8, 16, and 32 kHz tone bursts at varying intensity were given to evoke the ABRs of subject mice. To determine ABR thresholds, sound-pressure levels (SPL) between 15 and 70 dB SPL were used. Response signals were detected using subcutaneous needle electrodes.

### 4.3. Vestibular Evaluations

For vestibular evaluations, the mice were subjected to a series of tests (all performed at 8 weeks of age), including a swimming test and the rotarod test. For the swimming test, the swimming performance of mice was scored from 0 to 3, with 0 being normal swimming and 3 being underwater tumbling [40]. For the rotarod test, mice were placed on a rotating rod for a maximum of 240 s. The speed of the rod’s rotation was accelerated from 5 rpm to the maximal speed of 20 rpm for 1 min. The duration for which each mouse was able to remain on the rotating rod was recorded [41].

### 4.4. Inner-Ear Histology Studies

To perform light-microscopy analysis, tissue samples were subjected to hematoxylin and eosin (H&E) staining, and the morphology of each sample was determined using a Leica optical microscope (Leica). First, inner-ear tissue from adult mice (3 months) was harvested and fixed using perilymphatic perfusion with 4% paraformaldehyde (Bio Basic Inc., Markham, ON, Canada), prepared in phosphate-buffered saline (PBS). Specimens were decalcified for 1 week. Subsequently, samples were dehydrated and embedded in paraffin. Serial sections of 7 µm thickness were obtained and stained with H&E.

For the whole-mount studies of mouse inner ears, specimens were prepared as previously described [16]. Tissue samples were stained with rabbit anti-Myosin-VIIA primary antibody (1:200; Proteus Bioscience Inc., Ramona, CA, USA). Later, tissue sections were incubated with DAPI (1:5000; Thermo Fisher, Waltham, MA, USA) and Alexa Fluor 568-conjugated goat antirabbit IgG (H + L) secondary antibodies (1:200; Thermo Fisher, Waltham, MA, USA) at 4 °C overnight. After washing with PBS to remove any residual antibodies, tissue samples were mounted using the ProLong Antifade Kit (Molecular Probes, Eugene, OR, USA) for 20 min at room temperature. Images of mice inner-ear tissue samples were captured using a laser-scanning confocal microscope (LSM880, Zeiss, Oberkochen, Germany).

### 4.5. Pendrin Expression

For pendrin-expression analysis, tissue sections were harvested from the inner ears of *Slc26a4^919-2A>G/C565Y^* and *Slc26a4^C565Y/C565Y^* mice. Tissue sections were mounted onto silane-coated glass slides, deparaffinized in xylene, and rehydrated in ethanol. Tissue samples were stained with rabbit antipendrin (1:1000; antibody kindly provided by Dr. Jinsei Jung, Yonsei University College of Medicine, Seoul, Korea), and incubated with DAPI (1:5000) and Alexa Fluor 488-conjugated goat antirabbit IgG (H + L) secondary antibodies (1:200; Thermo Fisher). After incubation, slides were washed with PBS and mounted with the ProLong Antifade Kit at 25 °C. Images were captured using a laser-scanning confocal microscope (LSM880, Zeiss).

### 4.6. Statistical Analyses

Data are presented as mean ± SD. Statistical analyses were conducted using unpaired Student’s t-test with Bonferroni correction for continuous variables. A *p* value < 0.05 indicates significance. All analyses were performed using SPSS/Windows software 15.0 (SPSS Inc., Chicago, IL, USA).

## Figures and Tables

**Table 1:** Comparison of phenotypes among mouse strains with different *Slc26a4* variants.

	*Slc26a4^−/−^* [13]	*Slc26a4^loop/loop^* [14]	Tg[E];Tg[R];*Slc26a4*^Δ/Δ^ [18]	*Slc26a4^919A>G/919A>G^* [15]	*Slc26a4^H723R/H723R^* [16]	Slc26a4 L236P [17]	hH723R Tg [19]	*Slc26a4^C565Y/C565Y^*
Audiological phenotypes	Profound hearing loss (>100 dB SPL)	Profound hearing loss (>100 dB SPL)	Hearing levels depend on the time of *Slc26a4* expression. Doxycycline initiation at E18.5 (IE18.5) results in partial hearing loss	Profound hearing loss (>120 dB SPL)	Normal	Moderate-to-profound hearing loss in mice at 1 month. No progressive hearing loss up to 9 months	Profound hearing loss (>100 dB SPL)	Normal
Cochlear hair cells	Severe degeneration of inner and outer hair cells by P45	ND	Functionally intact at P25 to P35	Severe degeneration of inner and outer hair cells at 6 w	Normal up to P60	Different degrees of hair-cell degeneration and abnormal structures of stereocilia	Mild-to-severe degeneration of hair cells	Normal up to P90
Stria vascularis	Atrophic	ND	No significant difference between wild-type, IE18.5, and discontinued at E17.5 (DE17.5)	Atrophic	Normal	Atrophic	Atrophic	Normal
Vestibular aqueduct and enndolymphatic hydrops	Enlarged	ND	Size depends on time of Slc26a4 expression. Significantly enlarged in E18.5 mice	Enlarged	Normal	ND	Enlarged	Normal
Vestibular phenotypes	Vestibular deficits, including head tilting, head bobbing, and circling	Variable vestibular deficits, including unsteady gait, circling and tilted body	ND	46% of mice with head tilting and circling	Normal	9 of 31 L236P mice had balance dysfunction. Vestibular dysfunction variable in L236P mice	ND	Normal
Vestibular hair cells	Severe degeneration of vestibular hair cells by P45	Normal morphology of vestibular hair cells at 2 m	ND	Loss and degeneration of utricular hair cells	Normal	Normal	ND	Normal

ND, not described. Note: *Slc26a4^loop/loop^*, mice homozygous for p.S408F mutation.

## Supplementary Materials

The following are available online at https://www.mdpi.com/1422-0067/22/6/2789/s1, Figure S1: Auditory tracking up to 44 weeks, Table S1: Criteria of assessment for pathogenic variants and verdicts for SLC26A4: p.C565Y from Varsome platform, Table S2: The list of explanations for corresponding criteria for SLC26A4: p.C565Y from Varsome platform, Table S3: Classification rule for various combination of multiple ACMG criteria and pathogenicity verdicts for SLC26A4: p.C565Y.
