# Supplementary material for: Toward the Pathogenicity of the SLC26A4 p.C565Y Variant Using a Genetically Driven Mouse Model
Source: Int J Mol Sci. 2021 Mar 10;22(6):2789. doi: 10.3390/ijms22062789 (PMC8001573; doi:10.3390/ijms22062789)
Supplement: Supplementary file 1 [file ijms-22-02789-s001.zip › ijms-1114824-proofreading done-supp/supplementary tables.pdf]

Toward the pathogenicity of the *SLC26A4* p.C565Y variant using a genetically driven mouse model

Chin-Ju Hu[1,#], Ying-Chang Lu[1,2,#], Ting-Hua Yang[1], Yen-Hui Chan[1,7], Cheng-Yu Tsai[1], I-Shing Yu[6], Shu-Wha Lin[6], Tien-Chen Liu[1], Yen-Fu Cheng[2,3,4*], Chen-Chi Wu[1,5,8,*], Chuan-Jen Hsu[1,7]

(1) Department of Otolaryngology, National Taiwan University Hospital, Taipei, 100, Taiwan

(2) Department of Medical Research, Taipei Veteran General Hospital, Taipei, 112, Taiwan

(3) Department of Otolaryngology-Head and Neck Surgery, Taipei Veteran General Hospital, Taipei 112, Taiwan

(4) School of Medicine, National Yang-Ming University, Taipei 112, Taiwan

(5) Department of Medical Genetics, National Taiwan University Hospital, Taipei, 100, Taiwan

(6) Transgenic Mouse Models Core (TMMC), Division of Genomic Medicine, Research Center for Medical Excellence, National Taiwan University, Taipei, 100, Taiwan

(7) Department of Otolaryngology, Taichung Tzu Chi Hospital, Buddhist Tzu Chi

Medical Foundation, Taichung, 427, Taiwan

(8) Department of Otolaryngology, College of Medicine, National Taiwan University, Taipei, 100, Taiwan

Running title: Mouse model with *Slc26a4* p.C565Y variant

* Corresponding authors.

Address: Department of Medical Research, Taipei Veteran General Hospital, 201, Sec.2, Shi-Pai Rd., Taipei 112, Taiwan (Y-F. C.) & Department of Otolaryngology, National Taiwan University Hospital, 7 Chung-Shan S. Rd., Taipei, 100, Taiwan (C-C. W.).

E-mail: yfcheng2@vghtpe.gov.tw (Y-F. C.) & chenchiwu@ntuh.gov.tw (C-C. W.)

[#] The first two authors contribute equally to this article.

Supplementary Table 1. Criteria of assessment for pathogenic variants and verdicts for *SLC26A4*:p.C565Y from Varsome platform

	**Supporting (PP)**	**Moderate (PM)**	**Strong (PS)**	**Very Strong (PVS)**
**Allele frequency**		Absent in population database. ✓ **(PM2)**	Significantly increased prevalence in affected population compared to control group. **(PS4)**	
**Theoretical or predictive results**	Multiple predictive algorithms to support damages caused by variants. ✓ **(PP3)**	Novel missense change at reported pathogenic locus. ✓ **(PM5)**<br>Protein lengths change by in-frame insertion/deletion. **(PM4)**	Variants cause same reported pathogenic amino acid changes. **(PS1)**	Variants bring about null gene expression (e.g. abnormal splicing, termination, frameshifts). **(PVS1)**
**Functional evidence**	High rate of reported pathogenic missense variants in a gene are commonly involved in mechanism of the disease. ✓ **(PP2)**	Variants locating at hot spots or functional domain without recorded benign variation in the gene. ✓ **(PM1)**	Well-established functional studies as evidence for the deleterious effects caused by variants. **(PS3)**	
**Segregation extent**	Co-segregation of causative variants in multiple affected family members. **(PP1)**			
**De novo pathogenic mutation**		De novo mutation but not confirmed by paternity testing. **(PM6)**	De novo mutation and verified by paternity testing. **(PS2)**	
**In trans with pathogenic variants**		Detected trans-variant on opposing allelic locus of another pathogenic variant. **(PM3)**		
**Records from reputable database**	Same locus annotated as pathogenic variants in reputable database. ✓ **(PP5)**			
**Specific phenotype**	Symptoms in patients are high specific for a genetic etiology. **(PP4)**			

§ Above table modified from guideline criteria of American College of Medical Genetics and Genomics (ACMG)

Supplementary Table 2. The list of explanations for corresponding criteria for *SLC26A4*:p.C565Y from Varsome platform

Criteria	Explanation
PM1	Hot-spot of length 61 base-pairs has 11 non-VUS* coding variants (11 pathogenic and 0 benign), pathogenicity = 100.0%, qualifies as hot-spot.
PM2	Variant not found in gnomAD exomes (good gnomAD exomes coverage = 63.4).<br>GnomAD genomes homozygous allele count = 0 is less than 3 threshold for recessive gene *SLC26A4* (good gnomAD genomes coverage = 29.5).
PM5	Alternative variant chr7:107340606:T>G (Cys565Gly) is classified “Likely Pathogenic” with 1 star, by ClinVar (and confirmed using ACMG).
PP2	◆ 177 out of 194 non-VUS missense variants in gene *SLC26A4* are pathogenic = 91.2% which is more than threshold of 51.0%.<br>◆ 327 out of 593 clinically reported variants in gene *SLC26A4* are pathogenic = 55.1% which is more than threshold of 12.0%.
PP3	Pathogenic computational verdict based on 7 pathogenic predictions (DANN, FATHMM-MKL, M-CAP, MVP, MutationTaster, REVEL and SIFT) vs 4 benign predictions (DEOGEN2, EIGEN, MutationAssessor and PrimateAI).
PP5	◆ ClinVar classifies this variant as “Likely Pathogenic”, rated 2 stars, with 3 submissions, 16 publications and no conflicts.<br>◆ UniProt classifies this variant as “Pathogenic”, associated with Pendred syndrome (PS) and rare genetic deafness. Its related publications: PMID 14679580, 19204907 and 9618166.

§ All the above contents are quoted from Varsome platform (https://varsome.com/). (Data quated at 2020/07/10)

* VUS: variants of uncertain significance.

Supplementary Table 3 Classification rule for various combination of multiple ACMG criteria and pathogenicity verdicts for *SLC26A4*:p.C565Y

Pathogenicity verdict	Combination of various ACMG criteria			
Pathogenic	(1)	1 PVS1	And	≥1 PS<br>≥2 PM<br>1 PM & 1 PP<br>≥2 PP
	(2)	≥2 PS		
	(3)	1 PS	And	≥ 3 PM<br>2 PM & ≥2 PP<br>1 PM & ≥4 PP
Likely Pathogenic	(1)	1 PVS1 & 1 PM		
	(2)	1 PS & 1-2 PM		
	(3)	1 PS & ≥2 PP		
	(4)	≥3 PM (for *SLC26A4*:p.C565[illegible] ✔		
	(5)	2 PM & ≥2 PP		
	(6)	1 PM & ≥4 PP		

§ Above table modified from guideline criteria of American College of Medical Genetics and Genomics (ACMG)

§ PVS: pathogenic very strong; PS: pathogenic Strong (PS1-PS4), PM: pathogenic Moderate (PM1-PM6); pathogenic supporting (PP1-PP5)
