# Supplementary figures and images for: Toward the Pathogenicity of the SLC26A4 p.C565Y Variant Using a Genetically Driven Mouse Model
Source: Int J Mol Sci. 2021 Mar 10;22(6):2789. doi: 10.3390/ijms22062789 (PMC8001573; doi:10.3390/ijms22062789)

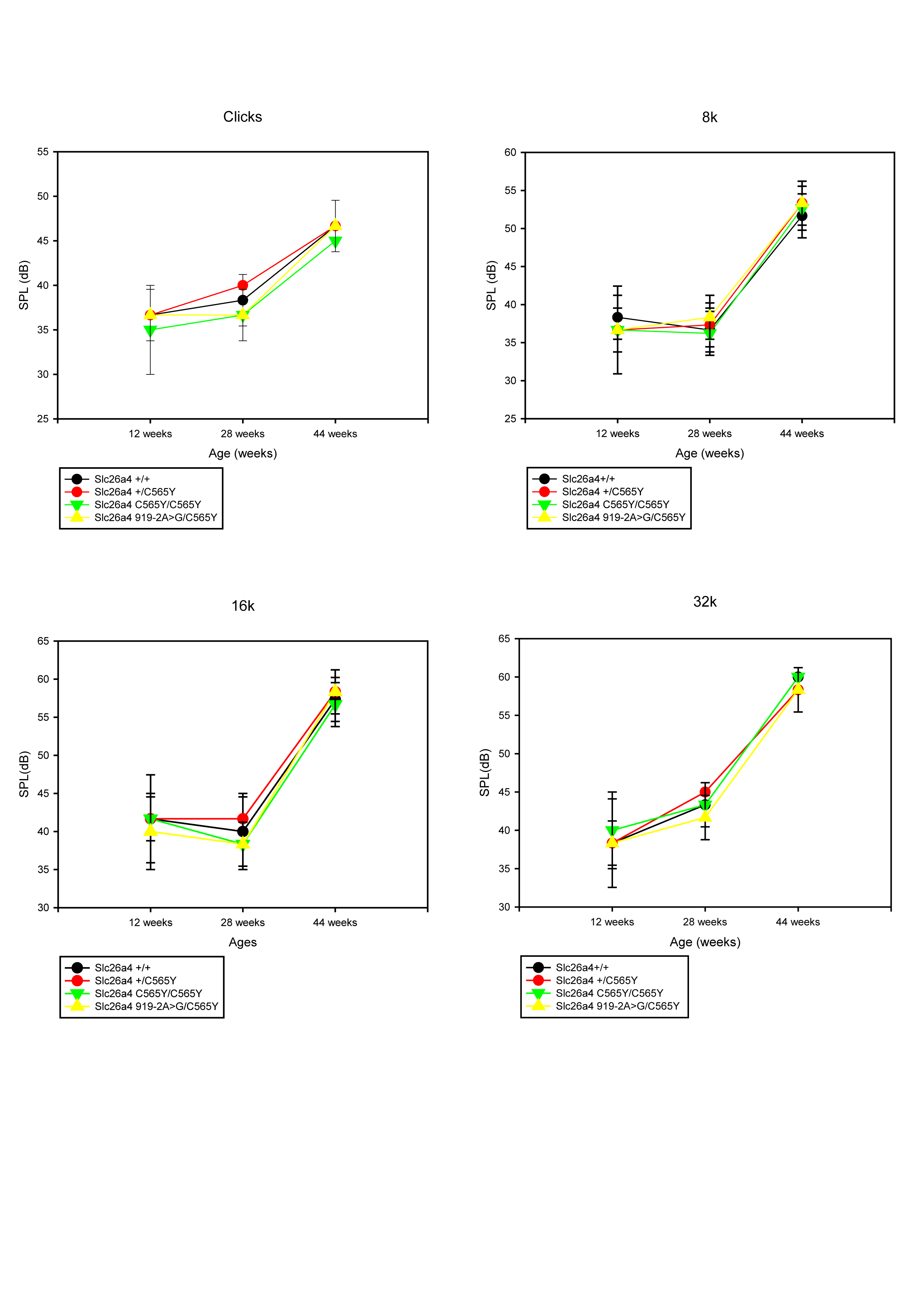

Supplement: Supplementary file 1 [file ijms-22-02789-s001.zip › ijms-1114824-proofreading done-supp/supplementary figure.tif]
